# High tibial osteotomy: A review of the readability and quality of patient information on the internet

**DOI:** 10.34172/hpp.2021.41

**Published:** 2021-08-18

**Authors:** Matthew Clark, Ian Colin Baxter, Matthew Hampton, Robert D Sandler, Andrew Legg

**Affiliations:** ^1^Department of Trauma and Orthopaedics, The Rotherham NHS Foundation Trust, Rotherham, United Kingdom; ^2^Department of Trauma and Orthopaedics, Sheffield Children’s Hospital, Sheffield, United Kingdom; ^3^Department of Rheumatology, The Rotherham NHS Foundation Trust, Rotherham, United Kingdom

**Keywords:** Health literacy, Comprehension, Internet Based intervention, Osteoarthritis, Osteotomy

## Abstract

**Background:** High tibial osteotomy (HTO) is a common procedure performed for unicompartmental knee osteoarthritis (OA). Patients are increasingly using the internet to research surgical procedures to help aid decision making. Our aim was to assess the readability and quality of information available to patients online relating to HTO.

**Methods:** A systematic review of three search engines Google®, Bing®, and Yahoo® using the search terms "high tibial osteotomy" and "tibial osteotomy" separately was performed. The first three pages of results for each search engine were analyzed. Readability was assessed using the Flesch Reading Ease Scale (FRES), Flesch-Kincaid Grade level (FKGL) and the Simple Measure of Gobbledygook formula (SMOG). Quality was assessed with the DISCERN questionnaire, JAMAbenchmarks and the presence of Health on the Net Foundation Code of Conduct (HONCode).

** Results:** Twenty-four webpages were included after duplicates (n=42) and exclusions (n=24).The overall readability was low, with a mean FRES of 53.2 (SD: 9.1), FKGL 10.7 (SD: 1.8),SMOG 10.4 (SD: 1.5). Quality was also low with a mean DISCERN score of 42 (SD: 12.3).None of the webpages fulfilled all of the JAMA benchmarking criteria and only 2/24 (8.3%)webpages possessed HONCode certification.

**Conclusion:** The overall online information available to patient’s considering HTO is of lowreadability and quality. Improving the quality and readability of patient information online willbenefit informed patient decision making before HTO surgery.

## Introduction

Knee osteoarthritis (OA) is the most common joint disorder, representing a significant health burden.^1^ The lifetime risk of developing knee OA is estimated at 47% in women and 40% in men.^2^ Symptoms of knee OA include pain, stiffness and activity limitations.

Initial management of knee OA is non-operative, with simple analgesics and lifestyle adaptations including weight loss and activity modification to relieve symptoms. If symptoms progress despite optimisation of non-operative measures, surgical options are considered. High tibial osteotomy (HTO) offers an alternative treatment to arthroplasty in active patients with malalignment and isolated compartment OA. HTO is a joint preserving procedure which allows return to high impact activities and sports with no restrictions,^3^ whilst delaying or preventing any subsequent requirement for arthroplasty.

Patient’s utilise many types of information when making a decision about elective surgery, including online information, leaflets, and face-to-face consultations.^4^ Information retention following a face-to-face consultation is low.^5^ A substantial proportion of patients therefore utilise the internet to research any potential surgery following their initial consultation.^4,6^ The internet is largely unregulated and there are concerns regarding the quality of information provided.^7,8^ Information presented to patients must be of high quality and appropriate readability to promote patient engagement with decision-making and prevent unnecessary anxiety, distress and mis-information.

Readability of text refers to the levels of reading difficulty based on sentence length and syllable count. Readability reflects the comprehension level a person must have to understand the health information presented. It is recommended that the readability of health care literature should be of US school grade level 5 or below equating to age 11 years.^9,10^ The usefulness of online material may also be limited if the information presented is of poor quality. This can misinform the patient and provide a bias picture of the treatment proposed. Previous studies have found information online related to orthopaedics to be of low quality.^11-13^

The patient’s ability to access information and interpret the information can impact the decisions they make about treatment going forward.^14^ Patient’s age and socioeconomic status can affect engagement with online information.^15^ It is important to enable shared decision making around healthcare decisions. In doing so patient’s engage more actively in follow up, which is vitally important following HTO.^16^ To support this shared patient decision making, information provided to patients needs to be clear, accessible, focussed and evidenced based as well as including risks and likely outcomes from each intervention.^17^

In this study, we aim to examine the information that is available on the internet for patients undergoing or considering HTO, for both quality and readability. This is the first study, to our knowledge, which looks at online information relating to HTO.

## Materials and Methods

A review of the online literature available to patient’s relating to HTO was conducted and the webpage data analysed.

**Figure 1:**
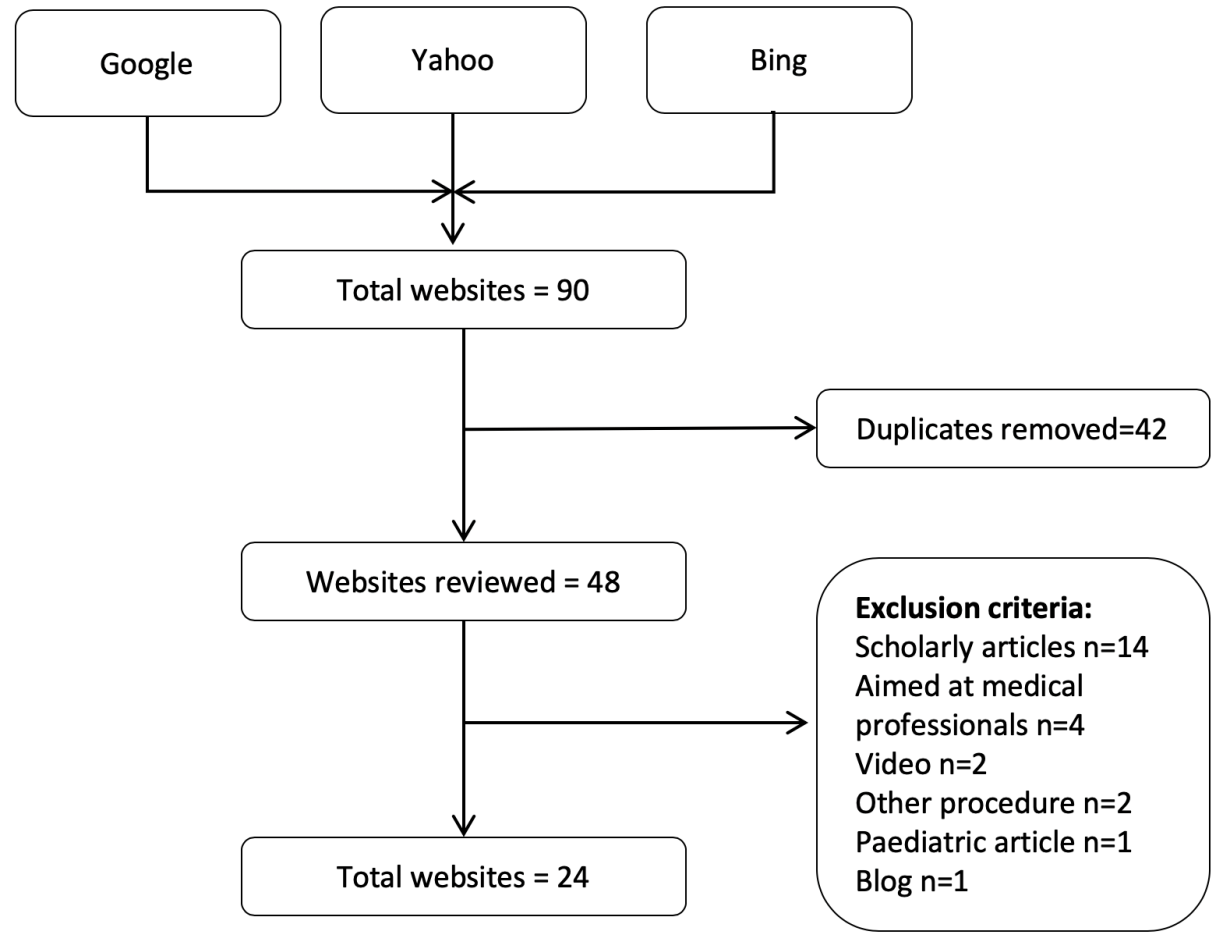
**Website inclusion process.** A total of 90 webpages were screened with 42 duplicates removed. 24 webpages were removed based on exclusion criteria with 24 webpages remaining for analysis

Two independent investigators (MC + MH) used Google®, Bing® and Yahoo® search engines to search the terms “high tibial osteotomy” and “tibial osteotomy” separately (April 2020). The first three pages of results for each search engine were reviewed. This strategy was chosen as internet users rarely go beyond the first three pages of results.^18^ Any duplicates were removed from subsequent analysis. Inclusion criteria included webpages related to HTO including, articles giving information that is relevant to patients undergoing or considering HTO (indications, techniques, recovery time and outcomes). Exclusion criteria included language other than in English, scholarly articles, advertisements for products and hospitals, personal experience or blogs, paediatric resources, video resources, password protected sites, articles not related to HTO and material solely aimed at medical professionals. A third investigator (IB) resolved any disagreement regarding the inclusion of web pages. The website title and address were recorded on a spreadsheet prior to full data extraction. Raw text was extracted from the webpages and stored. Videos and images were not examined (Figure 1).

The readability of each webpage was calculated using the Flesch Reading Ease Scale (FRES), Flesch-Kincaid Grade level (FKGL) and the Simple Measure of Gobbledygook formula (SMOG). A widely available free online tool was used to calculate these scores (https://readabilityformulas.com). Titles, subtitles, references and advertising text were excluded from the analysis, including only body text and bullet points in the analysis.

The FRES and the FKGL provide quantitative measures of readability. The FRES is calculated from the formula 206.835 – 1.015 x (average number of words per sentence) – 84.6 x (average number of syllables per word). It scores the text from 0-100, with a high score representing easier to read text. The FKGL is calculated from 0.39 x (words/sentences) + 11.8 x (syllables/words) – 15.59. The FKGL estimates the US school grade reading ability required to understand the text.

The SMOG readability formula estimates the number of years of education a person requires to understand a piece of written information. In brief, this is calculated from the square root of the number of words with more than three syllables in a sample of 10 sentences at the beginning, middle and end of the text, and adding 3.

### DISCERN tool

The DISCERN tool is a validated and reliable instrument for assessing the quality of written healthcare information related to treatment choices.^17^ The DISCERN questionnaire comprises of 16 questions analysing reliability and quality of information. Each question is scored on a Likert scale from 1-5. A score of 1 corresponds to a “no” answer, 3 to “partially” and 5 to “definite yes”. Section one, questions 1 to 8, focuses on the reliability of the source and section two, questions 9 to 15, focuses on the quality of the information provided on treatment choices. Question 16 is an overall judgement of the information. The maximum achievable score is 80.

Example questions examining reliability of the source include:

*“Is it (the source) clear what sources of information were used to compile the publication (other than the author or producer)?*

*Is it (the source) balanced and unbiased?”*

Example questions examining quality of the source include:

*“Does it describe how each treatment works?*

*Does it describe how the treatment choices affect overall quality of life?”*

Two independent investigators scored each webpage using the DISCERN tool. A disagreement in >2 points for the final score led to the webpage being scored by a third independent investigator. If the third reviewer was within 2 points of one of the original reviews, the mean score was taken of these two. If the third reviewer was >2 points from each of the two previous scores, a mean of the three scores was used.

### JAMA benchmarking

The JAMA benchmarks were established by the *Journal of the American Medical Association* (*JAMA*).^19^ This provides a score for a webpage from 0-4. The score is generated by scoring one point if each of the following are accounted for:

1. Authorship (authors/contributors and their affiliations and relevant credentials are provided)
2. Attribution (there are clear references and sources for all content included)
3. Disclosure (website ownership should be disclosed with sponsorship, commercial funding/support, conflicts of interest)
4. Currency (dates that the content was posted should be available and dates of any updates).

### Health On the Net certification

The Health on the Net Foundation Code of Conduct (HONcode), intends to hold adult health care literature web site developers to basic ethical standards in the presentation of information and help ensure readers always know the source and purpose of what they are reading.^20^ Website developers can apply to for a HONcode seal, if they can demonstrate they meet the eight principles of the HONcode. A HONcode seal is a marker of ethical editorial process, similar to JAMA benchmarking, but is not an endorsement of accuracy or reliability of information.

### Statistical analysis

The data were checked for normal distribution with the D’Agostino and Pearson normality test. Data are presented as median (inter-quartile range) for non-normally distributed data and mean (standard deviation) for normally distributed data. All analyses were completed on GraphPad Prism version 8.4.2 (San Diego, California, USA).

## Results

One hundred and eighty webpages were initially screened. After excluding duplicates between search engines and applying exclusion criteria, 24 webpages were included in the analysis.

### Readability

The mean FRES was 53.2 (standard deviation: 9.1), FKGL 10.7 (SD: 1.8), SMOG 10.4 (SD: 1.5). Only 4 webpages (16.6%) had a FRES greater than 60 and only 1 webpage (4%) had a FKGL of less than 8. The mean SMOG was 10.43 (SD: 1.50). None of the webpages had a recommended reading SMOG or FKGL of 5 or below.

### Quality

**Figure 2:**
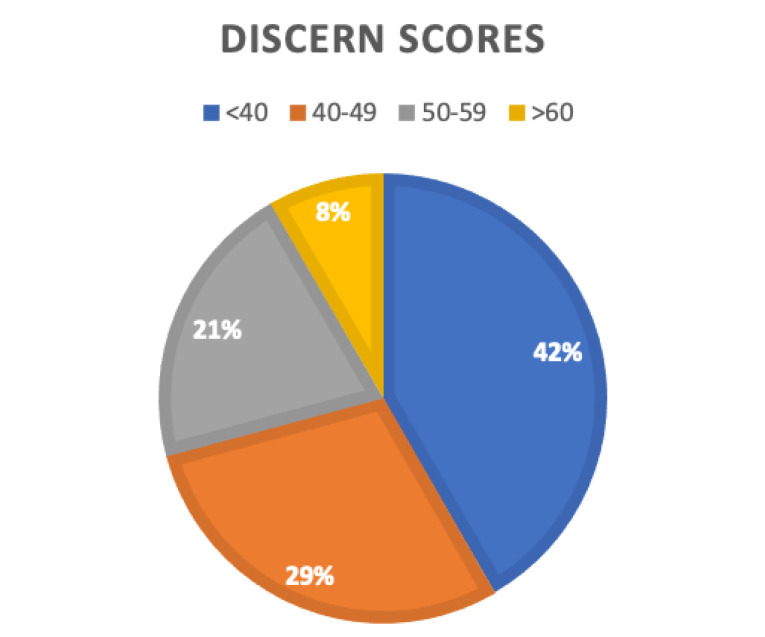
**
Pie chart to show break down of DISCERN scores.** Two webpages scored greater than 60 points on the DISCERN questionnaire (yellow), 5 webpages scored 50-59 points (grey), 7 webpages scored 40-49 points (orange) and 10 webpages scored less than 40 points (blue).

The mean total DISCERN score was 42.0 (SD: 12.3). Total DISCERN scores ranged from 23 to 63. Only 2 webpages (8.3%) had a DISCERN score of greater than 60, with 10/24 (41.7%) webpages scoring less than 40. The full breakdown of scores are displayed in Figure 2. Websites tended to score higher on descriptions of the treatment, the risks and the advantages. Websites scored poorly on the whole for having clear aims to the publication, clear sources of information, providing additional resources, describing what would happen if no treatment was given and providing support for shared decision making.

No websites met all four of the JAMA benchmarks. One website met three of the criteria, 8 met two of the criteria, 7 met one criterion and 8 webpages did not meet any of the criteria. The individual question results can be seen in Table 1. Two webpages had a health on the net seal of approval (2/24 8.3%).

**Table 1:** Breakdown of the number of online articles meeting each of the JAMA benchmarks

**JAMA Benchmark**	**Number of websites (%)**
Appropriate authorship	8/24 (33%)
Attribution of references	0/24 (0%)
Disclosure of funding/sponsorship	6/24 (25%)
Dates of content uploaded/updated	12/24 (50%)

## Discussion

In this study, we find the readability and quality of the information available online for patients to be of a low standard. To our knowledge, this is the first study to assess online patient information for HTO analysing both readability and quality measures. The prospect of any surgery can be daunting to patients. Many will use the internet to research the procedure prior to surgery, therefore ensuring information of good readability and quality online is of high importance.

The mean FRES in our study was 53.2 and the mean FKGL was 10.7, which is significantly higher than the US recommended FKGL of 5 or below.^9,10^ A readability study of information available online for ACL injuries analysing 35 web pages, found a FRES of 47.4 and FKGL 11.77^11^ and a study of patient information materials available on the American Association of Orthopaedic Surgery (AAOS) website found a FKGL of 10.43, with less than 2% of the resources having a readability of less than 6^th^ grade.^13^ A study analysing online patient materials for hip and knee joint arthroplasty reports a mean FKGL of 12, with only four articles (3%) having a FKGL of less than 8^th^ Grade.^21^ This is similar to our findings with only 4% of websites reporting a reading level below 8^th^ grade standard. Our readability scores showed a slightly easier level of comprehension than those reported in a study looking at the readability of stem cell therapy in the knee. The authors report a FRES of 35.9 and FKGL of 14. This is likely to be explained as the authors did not exclude academic articles. The results of the non-academic webpages are more consistent with our findings, with FRES ranging from 38.5-52.0.^12^ We excluded academic articles in our study to better represent patient experience online.

US guidelines recommend that health care literature should have readability of US grade reading level 5 or below to promote understanding amongst the general public.^9,10^ The mean SMOG reported in our study was 10.4. This is significantly higher than the fifth-grade target recommended. However, this value was lower than those reported in similar studies of readability in other medical conditions including Parkinson’s disease^22^ (mean SMOG 14.6), congestive heart failure^23^ (mean SMOG 11.39) and bariatric surgery (mean SMOG 12.43).^24^ Use of fewer words per sentence and fewer syllable words would help make the readability of the information easier online.

The DISCERN questionnaire has been used to examine online information available on stem cell injections in the knee. Ng et al reported a mean DISCERN score of 49.5 for all webpages, including academic articles.^12^ Our mean DISCERN score of 42 is lower than this as we did not include scholarly articles. Our mean DISCERN score is similar to other procedural based studies which excluded academic articles, for example endoscopic retrograde cholangiopancreatography (mean score 42)^25^ and caesarean section (mean score 43.6).^26^ Only two webpages included in our study had HONCode approval (8%) which is similar to a previous orthopaedic readability study on joint arthroplasty (12%).^27^

To help improve readability and quality of information relating to HTO online to promote the information delivered to patients we make the following recommendations. To improve the quality of information, websites should include clear authorship and potential conflicts of interests as well as a clear date of when the information is published. Websites generally performed well when describing HTO and the risks but seldom discussed alternative treatment options or what would happen without HTO. Additionally, including references and the source of the information presented and signposting to further information would help patients read further if they wished. To improve readability, we would encourage website authors to check their content on free online readability checkers, such as those used in this work, to understand the current grade reading age (FKGL or SMOG). If the authors recognise that this level is above the recommendation of 5, then rephrasing and simplifying the content would be recommended. Use of short sentences, and avoiding jargon, will help ensure patients do not misinterpret the information, which could lead to distress or anxiety around HTO. Addressing these issues would help promote patient education and better inform shared decision making outside the consulting room.

Our study has a number of strengths. We examined readability using three methods and quality using the DISCERN questionnaire, JAMA benchmarks and the presence of a HONCode seal. We excluded academic articles to better represent websites which patients are likely to access. There are limitations to this study. Firstly, search engines are dynamic and constantly changing. Therefore, sites may change going forward as more information about HTO becomes available. The accuracy of information presented was not assessed in this study. Furthermore, there are limitations to all current readability measures, with significant miscorrelation between formulae, though by using three separate measures, this mitigates the potential bias of using a single measure.

## Conclusions

The online information available to patients considering HTO is generally of low readability and quality. A greater awareness of readability and quality, and how online content creators can use freely-available validated tools and measures to actively quantify these, would encourage improvements, which will help ensure patients’ can access more appropriate information to inform shared decision making. Clinicians should also be aware of issues around readability and quality when signposting patients to online resources.

## Funding

This research did not receive any specific grant from funding agencies in the public, commercial, or not-for-profit sectors.

## Competing interests

There are no conflicts of interest to declare.

## Ethical approval

Not applicable.

## Authors’ contributions

MC designed the study, collected data, performed data analysis and drafted the manuscript. IB collected data and drafted the manuscript. MH collected data and drafted the manuscript. RDS designed the study, collected data and drafted the manuscript. AL coordinated the study and helped to draft the manuscript. All authors read and approved the final manuscript.

The authors claim that no part of this paper is copied from other sources.
